# Evaluation by 3D stereophotogrammetry of facial changes in edentulous patients after rehabilitation

**DOI:** 10.1590/1678-7757-2021-0462

**Published:** 2021-12-20

**Authors:** Guilherme Hideki de Lima TOYOSHIMA, Maria Giulia Rezende PUCCIARELLI, Karin Hermana NEPPELENBROEK, Chiarella SFORZA, Márcio de MENEZES, Thaís Marchini OLIVEIRA, Simone SOARES

**Affiliations:** 1 Universidade de São Paulo Faculdade de Odontologia de Bauru Departamento de Prótese e Periodontia São Paulo Bauru Brasil Universidade de São Paulo, Faculdade de Odontologia de Bauru, Departamento de Prótese e Periodontia, São Paulo, Bauru, Brasil.; 2 Università degli Studi di Milano Department of Biomedical Sciences for Health Milan Italy Università degli Studi di Milano, Department of Biomedical Sciences for Health, Milan, Italy.; 3 Universidade do Estado do Amazonas Amazonas Manaus Brasil Universidade do Estado do Amazonas, Amazonas, Manaus, Brasil.; 4 Universidade de São Paulo Faculdade de Odontologia de Bauru Departamento de Odontopediatria, Ortodontia e Saúde Coletiva Bauru São Paulo Brasil Universidade de São Paulo, Faculdade de Odontologia de Bauru, Departamento de Odontopediatria, Ortodontia e Saúde Coletiva, Bauru, São Paulo, Brasil.

**Keywords:** Complete denture, Stereophotogrammetry, Mouth rehabilitation

## Abstract

To assess facial changes after oral rehabilitation with complete dentures (CDs) by 3D technology allows understanding the results of a treatment that changes facial proportions. Precise outcome parameters can improve decision making.

**Objective:**

This descriptive observational research aimed to assess facial changes in completely edentulous patients after oral rehabilitation with a CD by a 3D stereophotogrammetry system.

**Methodology:**

30 edentulous patients (7 men and 23 women), aged 50 to 75, were analyzed with stereophotogrammetry at 28 previously determined anthropometric landmarks, at 2 different times: T1, before treatment, and T2, after inserting the CDs. Images were analyzed with a specific software for linear and angular measurements. The paired t-test was used to compare timestamps (α=0.05).

**Results:**

Major changes were observed in 7 of the 13 linear measures and 7 of the 9 angular measures. The following linear measurements had an increase: Sn-Gn (lower third of the face), Ls-Li (height of the vermilion lip), and ChL-ChR (mouth width). Sn-Ls (nasal philtrum height) decreased. For angular measurements, Sn-St-Pg (lower facial convexity) angles increased, and the Prn-Sn-Ls (nasolabial angle) and GoR-Pg-GoL (mandible convexity) angles decreased.

**Conclusions:**

Major facial changes in newly rehabilitated edentulous patients with CDs included an increase of the lower third of the face, of the vermilion lip, of mouth width, and of the lower facial convexity, and a decrease of the nasolabial angle and mandible convexity.

## Introduction

Complete edentulism in the older adults is a public health concern^1^ worldwide that varies with region. Edentulism rates have decreased steadily in developed countries, but they keep increasing in developing countries. Nevertheless, complete edentulism is growing due to an increased life expectancy.^2,3^ Many edentulous patients have social and psychological problems caused by difficulties in chewing, speaking, and socializing.^4,5^The etiology of edentulism involves can be iatrogenic, traumatic, or therapeutic causes. Periodontal disease, poor oral health, a high-sugar diet, and lower income, and poor education contributing to edentulism.^1^

Rehabilitation can be challenging for edentulous patients. Treatment must replace the teeth and the lost facial tissues in their function and looks.^3^ It should also improve quality of life.^2,6^ Esthetics of complete dentures can be subjective,^7,8^ changing according to the dentist’s technique, experience, and social and esthetic standards. Therefore, an esthetic facial reconstruction should predict possible facial changes before the treatment.^9^ Three elements that add to facial attraction are the eyes, facial musculature, and oral cavity, the last two affected by tooth loss.^10^Loss of the residual alveolar ridge affects the face,^11,12^ causing deeper wrinkles and a droopy labial commissure.^9^ A collapse of the lower facial third causes disharmony and reduces the vertical dimension of occlusion.^13^ Tools such as OHIP-EDENT (Oral Health Impact Profile for Edentulous) and OHRQoL (Oral Health-Related Quality of Life) were used in reports of patient satisfaction after oral rehabilitation with complete denture (CDs)^14,15^to assess its influence on their quality of life.^16,17^ However, studies that assess and measure this influence objectively are still incipient.Stereophotogrammetry (SPG) has been widely used to assess facial esthetics.^18-20^ It helps with decision-making and allows estimating the increased volume and area in standard procedures that are still based on trial. SPG has been recommended, validated, and accepted for clinical and research purposes.^21-23^ Among two studies that used stereophotogrammetry, the most recent analyzed facial changes in Chinese people,^24^and the other aimed to predict facial changes with software and mathematical algorithms.^9^ However, research on soft tissue changes in edentulous patients is still incipient. Changes in the lower third of the face critically affect the design and supplies of complete dentures.

This study aimed to compare edentulous patients before and after rehabilitation with CDs and to assess linear and angle measurements by SPG. We established anthropometric landmarks on each patient’s face, expecting changes in the linear and angular measurements before and after CDs.

## Methodology

### Approval from the Research Ethics Committee

This descriptive and observational study was approved by the ethics committee of the institution where the study was done, under protocol number CAAE: 99721718.6.0000.5417, according to the ethical standards of the Declaration of Helsinki. All participants signed an informed consent form before being included in the study.

### Sample Selection

Sample size was estimated based on a pilot study according to the paired t-test. Considering a minimum relevant change of at least 2.2 mm in soft tissues (standard deviation: 3.98 mm) with 0.05 of influence and 0.80 of test power, 28 participants were estimated.

In total, 30 white South Americans of Latin and mixed ethnicity, aged 50 to 75, joined the study. They were in routine care at the educational institution where the study was done. The inclusion criteria was men and women who had been edentulous (both in the maxilla and in the mandible) for 5 to 15 years. All participants already had complete removable dentures that needed to be replaced. The exclusion criteria included those with edentulism in only one of the dental arches, with signs or symptoms of TMDs (temporomandibular disorders), inability to cooperate, apparent facial asymmetry or associated craniofacial abnormalities and/or recent extractions, and those who did not sign the consent form. CDs were manufactured in a clinic, under the supervision of two specialists in oral rehabilitation, and in a laboratory, by a dental laboratory technician. The study was conducted between 2019 and 2020.

### Anthropometric landmarks

**Figure 1:**
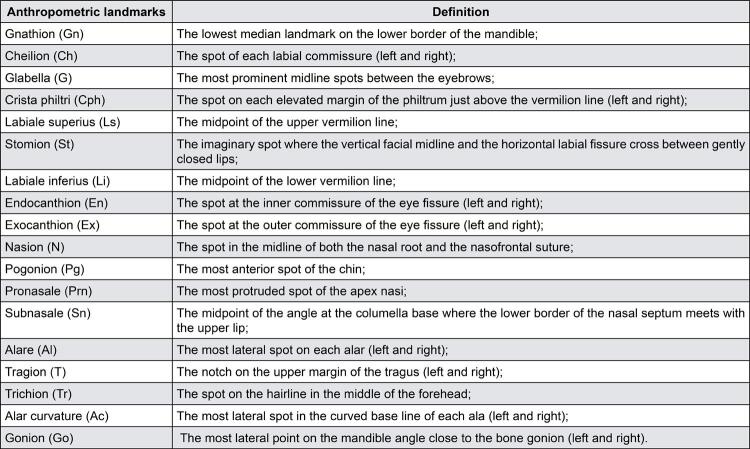
Anthropometric landmarks marked on the patient’s face for analysis

In total, 28 anthropometric landmarks (Figure 1) were marked once on the participants’ faces with an eyeliner (Make B., O Boticário, São José dos Pinhais, Paraná, Brazil) by an examiner for two pictures: before (T1) and after (T2) they had the CD. These landmarks were identified by two previously calibrated examiners. Landmark positioning had been previously tested and was satisfactory and reproducible.^25^

### Acquisition of 3D images (stereophotogrammetry)

Two calibrated operators acquired the stereophotogrammetry images using the portable system assisted by the VECTRA H1 camera (Canfield Scientific, Inc. Parsippany, New Jersey, USA). This method was reported as reliable and accurate.^26^ This system captures three images with a few seconds in between under specific conditions suggested by the manufacturer: for the first capture, the camera was placed at 45° to the participant’s right and about 20–30 cm below their face; the second capture was taken in frontal position; and for the third capture, the camera was placed at 45° to the left, similar to the first capture. All procedures followed the manufacturer’s guidelines.^27^ Once the pictures were taken, the device was connected to a laptop to assess the accuracy of 3D reconstructions automatically using the FACE SCULPTOR software (Canfield Scientific, Inc. Parsippany, New Jersey, USA).

Patients were instructed to remain seated and to look at a fixed spot before a sequence of pictures were taken. They had to remove earrings and to wear a cap to expose the front and the external auditory meatus.

Images were captured by stereophotogrammetry twice on the same day so the participants could be marked with anthropometric landmarks only once to reduce bias between the timestamps. In the first timestamp (T1), patients were in initial condition (edentulous without CDs); in the second timestamp (T2), they had CDs, with restored esthetics, function, and phonetics.

### Image analysis

**Figure 2:**
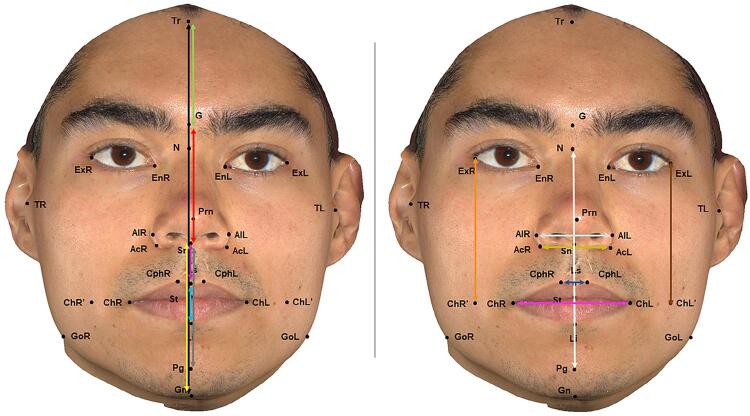
Linear measures obtained with the Vectra Analysis Module software (VAM elaboration, Canfield Scientific Inc.). Vertical lines – black: total facial height; green: upper third of the face; red: middle third of the face; yellow: lower third of the face; gray: anterior lower facial height; orange: distance between the exocanthion and the right cheilion; brown: distance between the exocanthion and the left cheilion; white: middle facial height. Horizontal lines – gold: width of the alar base; silver: width of the nose; navy-blue: width of the philtrum, and pink: mouth width

**Figure 3:**
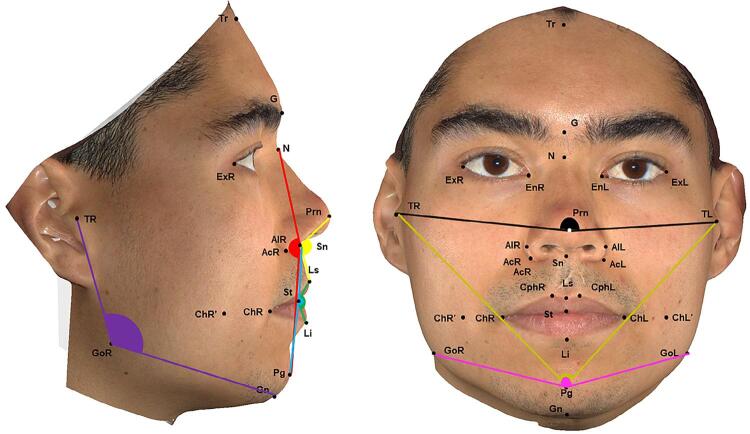
Angular measures obtained with the Vectra Analysis Module software (VAM elaboration, Canfield Scientific Inc.). Purple: right gonial angle; red: facial convexity (excluding nose); yellow: nasolabial angle; green: sealed lip angle; blue: lower facial convexity; black: middle facial convexity; gold: lower facial convexity; pink: mandibular convexity

**Figure 4:**
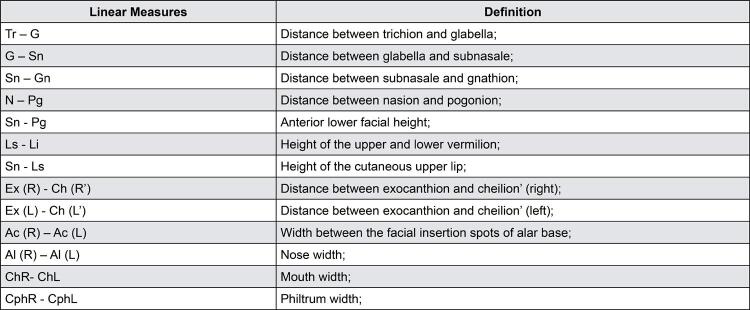
List of abbreviations and definition of linear measures Abbreviations: L: left side; R: right side.

**Figure 5:**
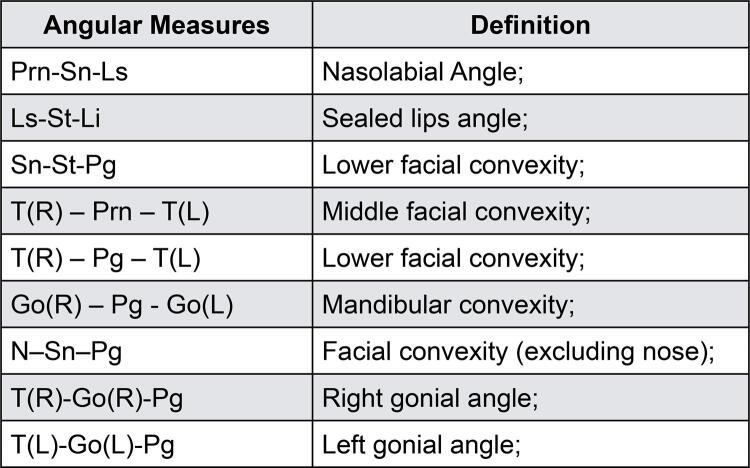
List of abbreviations and description of angles formed by linear measures Abbreviations: L: left side; R: right side.

The 28 morphometric points on the patient’s face were identified by a previously calibrated operator with the Vectra Analysis Module software (VAM elaboration, Canfield Scientific Inc., Parsippany, New Jersey, USA). After identification, the linear measurements (Figure 2) and angular measurements (Figure 3) described in Figure 4 and Figure 5 were also obtained.

**Figure 6:**
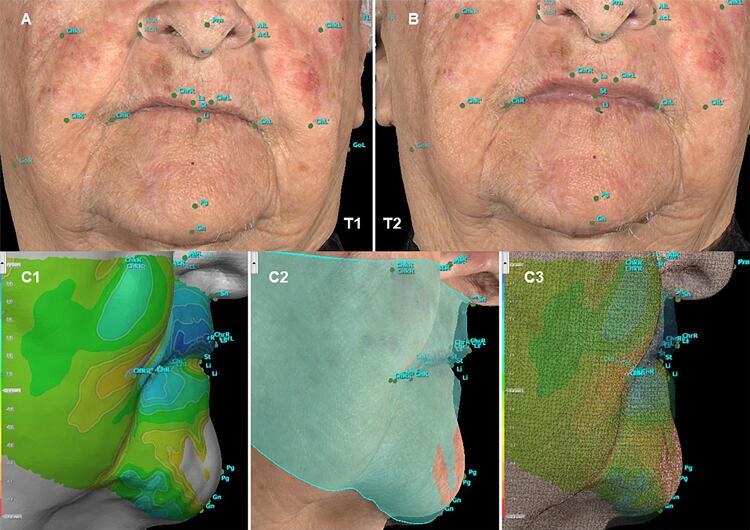
Facial changes between (A) T1 and (B) T2; (C1, C2, C3). Elaboration of a chromatic facial analysis with different colors (red: areas with -1 mm to 0 mm difference, green: 0 mm superimposed areas, and blue: areas with 0 to 2 mm difference) of the lower third of the face on sagittal view

Analysis was based on the evaluation of facial metrics following the anthropometric landmarks described by Farkas in 1994.^28^ Linear and angular measures were taken to determine noticeable facial changes after oral rehabilitation. This method was used to visibly assess facial changes, especially of the lower third of the face, since prostheses were expected to restore facial esthetics greatly (Figure 6).

**Figure 7:**
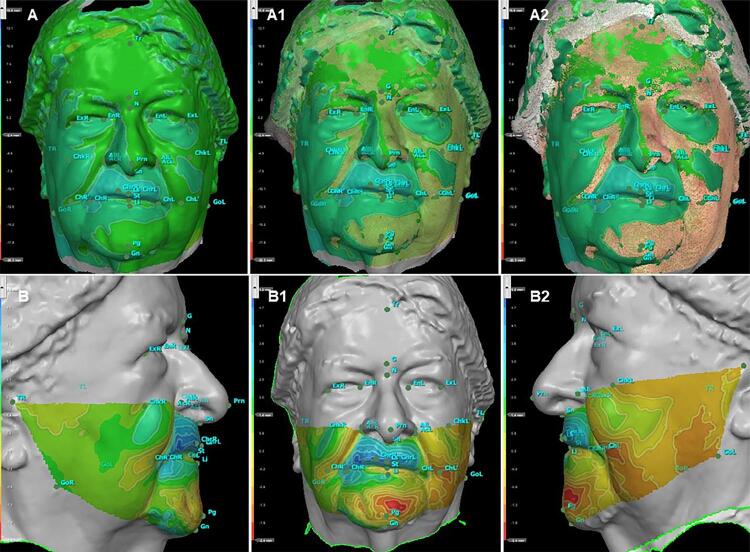
Steps of a chromatic facial analysis with different colors (red: areas with -1 mm to 0 mm difference, green: 0mm superimposed areas, and blue: areas with 0 to 2 mm difference) between two scans (T1 and T2). Superimposed full face (A, A1, A2), and superimposed lower third face from the right (B), from the front (B1), and from the left (B2)

For image overlay, the software was used to achieve a satisfactory alignment between images from T1 and T2 (registration error 0.098 mm). The two 3D images were manually registered by marks in known and unchanged areas: the upper front (between Tr and N) and two spots in front of the tragus were used, one on the right and another on the left of participants (TR and TL), as a reference for the overlap. Facial analysis used different colors to measure all changes (full-face superimposition) and emphasized the lower third of the face, with major changes in the labial protrusion; blue areas indicated about 3 mm increase from T1 to T2 (Figure 7).

### Method error

Differences in the facial anthropometric landmarks made by two experienced examiners determined the interoperator calibration error. These differences were found in one third of the sample after 15 days between evaluations and subjected to the paired t-test and Dahlberg formula^29^ to check the systematic error and randomness between measurements (p≥0.05).

### Statistical analysis

The paired t-test was used to measure facial changes in all participants at two timestamps (T1 and T2), with 0.05 of influence and 0.80 of test power. The Minitab software version 19 (Minitab, Inc., State College, PA, USA) was used for all statistical analyses.

## Results

In total, 30 patients who rehabilitated with maxillary and mandibular CDs joined the study: 7 men and 23 women. The kappa coefficient assessed the interoperator agreement (0.82 for analog measurements and 0.84 for digital measurements). For linear and angular measurements, the interoperator calibration was subjected to the paired t-test and Dahlberg formula^29^ to check the systematic and random error between measurements, showing that both examiners were calibrated (p≥0.05).

### Comparative analysis of linear measurements (mm) in T1 (without CD) and T2 (with CD)

Linear measurements were analyzed objectively, and seven out of 13 showed statistically significant changes (p<0.05) (Table 1).

**Table 1:** Comparative analysis of linear measures (mm) in two timestamps, T1: without complete dentures and T2: with conventional maxillary and mandibular complete dentures in all participants (n=30)

Linear measures (13)	T1 (Mean±SD)	T2 (Mean±SD)	p≤0.05
Tr-G	54.35±8.54	54.51±8.60	0.300
G-Sn	63.036±5.153	62.836±5.294	0.200
Sn-Gn	64.568±4.802	66.137±4.694	0.003*
N-Pg	101.79±7.13	103.30±6.87	0.013*
Sn-Pg	51.210±5.284	52.868±4.931	0.003*
Ls-Li	7.770±2.547	10.411±2.070	<0.0001*
Sn-Ls	18.084±3.221	17.525±3.109	0.008*
ExR-Ch’R	65.78±5.70	66.58±5.57	0.062
ExL-Ch’L	66.075±5.105	66.287±5.464	0.599
ChR-ChL	44.16±5.66	49.66±4.40	<0.0001*
CphR-CphL	10.80±1.88	13.08±7.77	0.126
AcR-AcL	36.170±3.827	36.680±3.981	0.040*
AlR-AlL	35.483±4.087	36.161±4.808	0.095

Paired t-test, * Statistically significant difference; SD: standard deviation

### Comparative analysis of angular measurements (degrees) in T1 (without CD) and T2 (with CD)

Angular measurements were made from angles formed by three distinct craniometric spots, and seven out of the 9 measures showed statistically significant changes (p<0.05) (Table 2).

**Table 2:** Comparative analysis of angles (degrees) in both timestamps, T1: without complete dentures and T2: with conventional maxillary and mandibular complete dentures in all participants (n=30)

Angular Measures (9)	T1 (Mean±SD)	T2 (Mean±SD)	p≤0.05
Prn-Sn-Ls	131.61±11.63	124.46±10.75	<0.0001*
Ls-St-Li	121.6±20.1	120.11±16.72	0.636
Sn-St-Pg	157.01±12.46	166.52±9.00	<0.0001*
TR-Prn-TL	65.881±3.214	65.972±3.518	0.763
TR-Pg-TL	63.853±2.509	63.165±2.017	0.006*
GoR-Pg-GoL	76.285±4.982	75.463±5.116	0.023*
N-Sn-Pg	170.81±7.92	168.60±8.22	<0.0001*
TR-GoR-Pg	127.159±5.150	127.652±5.320	0.046*
TL-GoL-Pg	125.42±5.70	126.09±5.84	0.024*

Paired t-test, * Statistically significant difference, SD: standard deviation

## Discussion

This study measured facial changes by 3D stereophotogrammetry before and after conventional CDs were applied in edentulous patients. Our hypothesis was confirmed by changes before and after CDs were applied in seven of 13 linear measurements and in seven of 9 angular measurements.

### Changes promoted by rehabilitation

In dentate individuals, the vertical dimension of occlusion is established by contact between the maxillary and mandibular teeth. Edentulism changes this vertical dimension of occlusion, which affects the lower third of the face and can cause an inappropriate facial shape, narrow upper and lower lip edges, and a contracted commissure. This makes the area seem “closed” and inverted.^30^ Sagittal facial analysis can show mandibular pseudo-prognathism and mandibular closure,^26,31^ which affect mastication and phonetics. Thus, changes in linear measurements showed that CDs strongly influenced the lower third of the face (Sn-Gn), the central facial height (N-Pg), the lower facial height (Sn-Pg), and the vertical dimension of occlusion.

Results for Sn-Pg were consistent with previous results^32,33^ in which such measures would increase after the prostheses were installed. However, Tartaglia, et al. evaluated complete-arch implant-supported prostheses.^33^ In our study, the lower third of the face (Sn-Gn) greatly increased, consistent with other studies.^33,24^Women’s lips grew significantly in Ls-Li (distance between upper and lower vermilion), since aging and decreased collagen synthesis cause tissues to lose elasticity and mobility.^34,35^ Ushijima, et al.^36^ (2013) confirmed changes in the upper and lower lips, especially when the vertical dimension of occlusion either decreased or increased, showing that the new prosthesis helps adjust lip position with bigger linear measurements, as seen in this study (T1=7.70±2.54; T2=10.41±2.07). Menezes, et al.^37^ (2011) reported that, in a group of patients aged 45 to 65, lips were significantly less thick, even in dentate individuals with at least 24 teeth, indicating that aging causes tissues to become less elastic and flabbier.

The lip philtrum (Sn-Ls) became smaller because, after inserting a maxillary CD, the base of the nose (Sn) and the upper limit of the upper lip (Ls) got closer, resulting in smaller measurements.

Mouth width (ChR-ChL) increased horizontally, showing that CDs can support the orbicularis musculature by changing the nasolabial sulcus, therefore returning traces of youthfulness. Our results (44.16±5.66 in T1 and 49.66±4.40 in T2) were incompatible with those of Tartaglia, et al.^33^(2012), who reported a large increase in vertical dimensions with no changes in mouth width. However, their study was based on implant-supported complete-arch prostheses with no buccal flange to support lip musculature.^33^ Major changes in linear measurements indicated that the degree of lip support affects not only the lip contour, but also the shape of the nasal base. This is compatible with the results of Fanibunda, Allcock and Thomason (2002), who reported a larger displacement of the upper and lower lips after inserting CDs and the dominance of maxillary CDs.^38^

Width between the facial insertions of the alae of the nose (AcR-AcL) increased with the CDs, since the prosthesis supports both the upper lip and the alae of the nose. However, the base of the nose increased in width with no statistical difference; it was expected to decrease with support, along with the tissue and nose rises. Linear measurements that were stable throughout rehabilitation, such as ExR-Ch’R and ExL-Ch’L, showed how these anatomic structures are important in recording the vertical dimension of rest to obtain the vertical dimension of occlusion in edentulous patients. Dentists and prosthodontists find it challenging to determine the vertical dimension of occlusion, since literature lacks a universally accepted scientific method.^39^ Helal and Hassan reported that in dentate patients, distance from the base of the chin (Gn) to the base of the nose (Sn) did not change compared to the distance from the exocanthion (ExR and ExL) to the corner of the mouth (ChR and ChL).^40^

In angular measurements, the nasolabial angle (Prn-Sn-Ls) was smaller with the CD (131.61°±11.63° in T1 and 124.46°±10.75° in T2) and had statistical relevance (p<0.0001), showing that this type of rehabilitation treatment supports tissue, same as in previous studies.^32,33^ Brunton and McCord^41^ (1993) reported that in dentate and edentulous individuals, average nasolabial angles were 109.67° and 96.20°, respectively, suggesting that facial harmony is between 95° and 110°. Lower facial convexity (Sn-St-Pg) was evaluated in the sagittal plane and greatly increased, as the teeth in CDs and the base of the prosthesis supported tissues around the mouth. Before treatment, these facial tissues were flaccid and less elastic, since they generally decrease due to aging.^42^ On the other hand, in the frontal plane, the lower horizontal facial convexity angle (TR-Pg-TL) decreased horizontally, therefore reducing angular measurements.

Convexity of the mandible (GoR-Pg-GoL) decreased after rehabilitation with conventional CD. Before rehabilitation, soft tissues were more spread out, but they showed greater support after treatment.

The angle of average facial convexity (N-Sn-Pg) decreased after the prosthesis was installed, showing that rehabilitation resulted in a more convex profile. Moreover, the greater the facial convexity, the smaller the angle between N-Sn-Pg, and the lower the convexity, the greater the angle.

The angles TL-GoL-Pg (left gonic angle) and TR-GoR-Pg (right gonic angle) were evaluated in the sagittal plane and increased, also showing that CDs can improve facial profile.

Facial implications of CDs regarding linear measurements include an increase of the lower third of the face because of bigger Sn-Gn and Sn-Pg. These changes imply harmonic facial thirds, with higher central facial height. CDs affected the space between the lips, reducing the labial philtrum and therefore the mouth (Ls-Li) and width (ChR-ChL), exposing labial tissue.

Most of the angular measurements decreased. CDs supported facial tissue because of a smaller facial convexity.

### Stereophotogrammetry

3D technology offers new possibilities for facial measurement, and stereophotogrammetry has proven to be reliable, valid, and reproducible. It allows accurate measuring and adjusts the vertical dimension, besides being more precise than calipers, used in some anthropometric studies.^19,24^

### Limitations

This study is limited regarding facial changes, since their esthetics are related to each dentist’s and patient’s sociocultural background. Besides, participants were all white, which limits the broadness of our results. The authors determined which ethnic group would be studied, since the participants come from a racially diverse country with many ethnicities.

## Conclusions

We found many facial changes in newly rehabilitated edentulous patients with CDs. The lower third of the face, the height of the vermilion lip, and mouth width increased with the CDs. The nasal philtrum became smaller. The lower facial convexity increased, while the nasolabial angle and mandible convexity decreased.
